# Cardiovascular Risk According to Plasma Apolipoprotein and Lipid Profiles in a Canadian First Nation* *This article is part of a joint publication initiative between *Preventing Chronic Disease* and *Chronic Diseases in Canada*. *Preventing Chronic Disease* is the primary publisher, while *Chronic Diseases in Canada* is the secondary publisher.


**Published:** 2010-12-15

**Authors:** Natalie D. Riediger, Sharon G. Bruce, T. Kue Young

**Affiliations:** University of Manitoba; University of Manitoba, Winnipeg, Manitoba, Canada; University of Toronto, Toronto, Ontario, Canada

## Abstract

**Introduction:**

Despite high diabetes rates among Canadian First Nations people, little is known about their cardiovascular disease risk. Our aim was to describe the apolipoprotein profile with respect to cardiovascular risk in a Canadian First Nation community.

**Methods:**

In 2003, a representative sample of adult members of a Manitoba First Nation (N = 483) participated in a screening study for diabetes and diabetes complications. We assessed their cardiovascular risk factors.

**Results:**

Sixty percent of women were at increased cardiovascular risk because of low apolipoprotein A1 (apoA1) levels, compared with 35% of men. The proportion of women with low apoA1 levels decreased with age, but the proportion with low high-density lipoprotein levels remained stable across age groups. Both apoB and apoA1 were significantly associated with obesity when age, sex, diastolic blood pressure, homocysteine, diabetes, and insulin resistance were controlled for.

**Conclusion:**

Apolipoprotein and lipid profiles in this First Nation population suggest high cardiovascular risk. Future research should characterize the lipoprotein particle size in this population.

## Introduction

In recent years, the prevalence of cardiovascular disease among Aboriginal people in Canada has been increasing and is now higher than the prevalence among non-Aboriginal people. In a random sample of Canadian Aboriginal people, the prevalence of cardiovascular disease was 18%, compared with 8% in Canadians of European descent (1).

Apolipoprotein A1 (apoA1) is the major protein of high-density lipoprotein (HDL), and apoB is among the major proteins of very low-, low- (LDL), and intermediate-density lipoproteins. Because of their associations with the respective lipoproteins, apoA1 is inversely and apoB is positively associated with cardiovascular risk (2). In fact, evidence suggests that apoA1 and apoB are better predictors of heart disease risk than are HDL and LDL cholesterol levels (3-5). Apolipoproteins may also offer advantages over lipoprotein cholesterol measurements because they are direct measurements, whereas LDL, for example, is calculated from other lipoproteins from a fasting blood sample.

Despite the high rate of diabetes and cardiovascular disease among Canadian Aboriginal people, little research has gone beyond examining traditional risk factors. In addition, research has been mostly based on chart review, and the few population-based studies that have been conducted have been limited to a single First Nation community (6-8). Our objective was to describe the apolipoprotein profile and its relationship to cardiovascular risk factors in a Canadian First Nation community.

## Methods

We conducted this study with data from a larger community-based screening study on diabetes complications (9). The sample of 483 men and women from a Manitoba First Nation was representative in terms of age and sex. Eligible participants (n = 1,356) were nonpregnant adults aged 18 years or older who were Registered Indians and lived in the community. The community is approximately 200 km northwest of Winnipeg, Manitoba. Data were collected from January through December 2003, and each participant had all of his or her data collected on the same day. Further details on the study can be found elsewhere (9). The study was approved by the University of Manitoba Health Research Ethics Board.

A registered nurse collected a fasting blood sample, and we assessed levels of plasma glucose, insulin, triglyceride, HDL cholesterol, LDL cholesterol, total cholesterol, total apoB, and apoA1. The nurse also measured blood pressure, urinary albumin and creatinine levels, and anthropometric characteristics (9). To assess insulin resistance, we used the homeostatic model assessment with glucose and insulin results in the following equation: (insulin [pmol] × 0.139) × (glucose [mmol/L]/22.5). Risk factors assessed in this study are defined in Table 1.

We analyzed all data by using SPSS version 16.0 for Windows (IBM, Chicago, Illinois). We compared plasma lipid levels by using *t* tests and Mann-Whitney U nonparametric tests. We compared differences in apolipoprotein levels by cardiovascular risk factors by using both *t* tests and Mann-Whitney U tests for variables with a nonnormal distribution or unequal variances. We used χ^2^ tests to detect differences in risk for cardiovascular disease by apolipoprotein category. Tests were 2-tailed, and *P* values <.05 were considered significant. To determine linear trends for mean apolipoprotein values by age group, we used 1-way analysis of variance with linear contrast. We estimated odds ratios for obesity by using backward stepwise multivariate logistic regression. We included variables in the model that were significantly associated with obesity in bivariate analyses. Those variables were age, sex, ever having smoked, systolic and diastolic blood pressure, presence of diabetes, triglyceride level, apoA1 level, apoB level, insulin resistance, homocysteine level, and microalbuminuria.

## Results

Risk for cardiovascular disease was high according to traditional cardiovascular risk factors such as HDL cholesterol and triglyceride levels (Table 2). Rates of obesity, diabetes, hypertension, and microalbuminuria in this population were also high (Table 3).

Significantly more women than men had apoA1 values that indicated cardiovascular risk (60% vs 35%; *P* < .001). Almost 18% of men and 12% of women had apoB values that indicated cardiovascular risk, but the difference was not significant. The proportion of participants with increased risk according to the apoB:apoA1 ratio was 54% for men and 57% for women, and the difference did not reach significance.

Mean apoB concentrations and the apoB:apoA1 ratio were significantly higher in men and participants with any cardiovascular risk factor (Table 3). Mean apoA1 concentrations were lower in patients with most cardiovascular risk factors, but the difference did not reach significance in patients with diabetes, hypertension, microalbuminuria, or cardiometabolic risk.

Cardiovascular risk tended to increase with age (Tables 4 and 5). We found a significant linear trend for age in men for apoB and apoB:apoA1 ratio. We also found a significant and positive linear trend for age in women for apoB and apoB:apoA1 ratio. Conversely, cardiovascular risk according to apoA1 decreased with age among women, and mean apoA1 levels increased with age. HDL cholesterol levels did not significantly increase or decrease with age among women (data not shown).

The final logistic model for presence of obesity included age, sex, diastolic blood pressure, diabetes, homocysteine, insulin resistance, apoA1, and apoB. A person with an apoA1 value of 1.14 g/L was 1.2 times as likely to be obese as was a person with an apoA1 of 1.20 g/L. Furthermore, the odds of obesity were 1.35 times as high for a person with an apoB level of 1.00 g/L as for a person with a level of 0.80 g/L.

## Discussion

According to plasma lipid levels and apolipoprotein profiles, the risk for cardiovascular disease is high among Canadian First Nations people. The abnormal plasma apolipoprotein concentrations we found are consistent with the high prevalence of obesity and diabetes in the community. Generally, participants had low HDL cholesterol, low apoA1, and high triglyceride levels, which typically coexist in people with insulin resistance.

The average lipid profile in the study community differed dramatically from that of the US population in general (11), most likely because of the high prevalence of diabetes in the community. For example, mean plasma LDL cholesterol levels among study participants were lower than those among NHANES participants for both sexes. However, plasma HDL cholesterol levels were lower and plasma triglyceride levels were much higher among our study participants than among the general American population. Compared with Australian Aboriginal people and Torres Strait Islanders (12), our study participants had slightly lower triglyceride and higher HDL cholesterol levels. However, these levels are still worse than those among other Canadian Cree-Ojibwa peoples, Inuit, or non-Aboriginal people, as assessed in the early 1990s (13). In addition, triglyceride and HDL cholesterol levels were higher and LDL cholesterol levels were lower in the study community than among another Canadian Aboriginal community (1); the other Aboriginal community, however, included only people aged 35 to 75 years, whereas our study sample included people aged 18 years or older, and the mean age was 38 years. Neither our lipid values nor the others described in this paragraph were age- or sex-standardized.

Paradoxically, the proportion of women with low apoA1 levels (higher cardiovascular risk) significantly decreased with age, and mean apoA1 levels had a significant positive linear trend with older age. The change in apoA1 levels by age in women seems to indicate more dyslipidemia in younger women. However, the proportion of women with HDL cholesterol levels that indicated increased risk remained stable across age groups. Therefore, for the same HDL cholesterol levels, older women (≥50 y) had lower apoA1 levels than did younger women (<50 years). This phenomenon may reflect a shift in HDL particle size in the older age groups, in which the proportion of small, dense HDL particles increases relative to large HDL particles. This explanation is further supported by the fact that the prevalence of diabetes increased with increasing age in our study.

Despite a likely genetic susceptibility to diabetes and its comorbidities in this First Nation community, we hypothesize that much of the dyslipidemia can be attributed to poor diet and inactivity. In the past, other indigenous communities, such as the Greenland Inuit, actually had more favorable lipid profiles than did nonindigenous people, most likely because of their traditional lifestyle (14). When compared with Danish controls, the Inuit had significantly higher apoA1 and significantly lower apoB, LDL cholesterol, total cholesterol, and triglyceride levels. Although apoA1 levels were significantly higher among the Inuit, HDL cholesterol levels were not, which indicates potential differences in the types of HDL between the 2 groups (differences in particle size). The Inuit may have a disproportionate number of atherogenic small, dense HDL particles, as opposed to the more beneficial large HDL particles, which is what we hypothesize in our study community, particularly among women. In a Canadian Oji-Cree First Nation community, although apoA1 levels were significantly lower among men with hypertriglyceridemic waist, apoA1 levels were actually nonsignificantly higher among women with hypertriglyceridemic waist (15). This apoA1 sex difference may partially explain the higher risk for coronary heart disease for women with diabetes than men with diabetes (16).

A preponderance of small, dense LDL and small HDL particles is associated with obesity (17), increased risk of coronary artery disease (18), and insulin resistance, regardless of diabetes status (19). Low HDL cholesterol concentrations among people with diabetes may indicate a specific reduction in large HDL particles (as well as a possible increase in small HDL particles), which may not necessarily significantly reduce apoA1 levels (19). In our study, because of the high rate of diabetes, we suspect that the low HDL cholesterol levels reported are due to the loss of large HDL particles, especially among older women. Women may also have lower HDL cholesterol levels than men in response to obesity (20), so apoA1 levels may also differ between men and women in response to obesity.

The apparent conflicting results of apoA1 and HDL cholesterol levels may affect the predictive nature of apoA1 and the apoB:apoA1 ratio for cardiovascular risk in this population. Future research should determine the association of apoA1 and apoB:apoA1 ratio on cardiovascular outcomes in First Nations peoples with diabetes. The apoB:apoA1 ratio was found to predict metabolic syndrome in nonobese but not obese participants (21), perhaps because obese participants are more likely to have diabetes. Mean apoA1 values were virtually identical in our study community, regardless of diabetes status, but mean HDL cholesterol levels were significantly lower in participants with diabetes. This finding supports the notion of an HDL profile of mostly small HDL particles in people in the community, which would keep plasma HDL levels low while increasing apoA1 levels.

Our study has several limitations. Although we cannot say whether the sample was completely representative of the population, it was representative according to age and sex and did not consist of those in the poorest health. Only 105 of the 275 community members with previously diagnosed diabetes participated. In addition, 3 of 10 community members with amputations and none of 15 members with end-stage renal disease participated. We also did not assess lipoprotein particle size and the distribution of sizes among the various types of lipoproteins. Finally, because of the cross-sectional nature of the data, no outcome data are available.

In conclusion, this community is at high cardiovascular risk, according to both plasma lipid and apolipoprotein profiles. Much of this risk is mediated by the high number of community members with diabetes and obesity and the associated changes in lipoprotein profile. More data regarding lipoprotein particle size and the distribution of small, medium, and large HDL and LDL particles are needed to confirm our hypotheses, but these preliminary data can be used to guide interventions that reduce the prevalence of chronic disease in Canadian First Nations peoples.

## Figures and Tables

**Table 1:** Risk Factors Assessed in a Study of Cardiovascular Risk in a Canadian First Nation, 2003

Risk Factor	Definition		

	Men		Women
Obesity	BMI ≥30.0 kg/m^2^		
High-risk WC	WC >102 cm		WC >88 cm
Diabetes	Self-report diagnosis, taking an oral hypoglycemic agent, or fasting glucose ≥7.0 mmol/L		
Hypertension	Self-report of diagnosis, SBP >140 mm Hg, or DBP >90 mm Hg		
Dyslipidemia	Fasting plasma TG ≥1.7 mmol/L and fasting plasma HDL cholesterol ≤1.03 mmol/L	Fasting plasma TG ≥1.7 mmol/L and fasting plasma HDL cholesterol ≤1.3 mmol/L	
Microalbuminuria^a^	ACR >2.0 mg/mmol	ACR >2.8 mg/mmol	
Metabolic syndrome	Adult Treatment Panel III criteria (10)		
Cardiometabolic risk	At-risk WC plus plasma TG ≥1.7 mmol/L		
Low apoA1	ApoA1 <1.07 g/L	ApoA1 <1.22 g/L	
High apoB	ApoB >1.2 g/L		
High apoB:apoA1 ratio (5)	>0.8	>0.7	

Abbreviations: BMI, body mass index; WC, waist circumference; SBP, systolic blood pressure; DBP, diastolic blood pressure; TG, triglyceride; HDL, high-density lipoprotein; ACR, albumin-to-creatinine ratio; apo, apolipoprotein.

a Determined by using the Bayer DCA 2000 point-of-care analyzer (Elkhart, Indiana).

**Table 2:** Plasma Lipid Levels Among 483 Canadian First Nations Adults, 2003

**Lipid^a^**	Men (n = 230), Mean (SD)	Women (n = 253), Mean (SD)	*P* Value^b^	Men and Women, Mean (SD)
Triglycerides, mmol/L	2.3 (2.5)	2.1 (2.0)	.86	2.2 (2.3)
	1.7 (1.1-2.6)^c^	1.7 (1.2-2.5)^c^		1.7 (1.2-2.6)^c^
LDL cholesterol, mmol/L	2.9 (0.9)	2.6 (0.9)	<.001	2.7 (0.9)
HDL cholesterol, mmol/L	1.2 (0.3)	1.2 (0.3)	<.04^d^	1.2 (0.3)
Total cholesterol, mmol/L	5.0 (1.2)	4.8 (1.1)	.07	4.9 (1.2)

Abbreviations: SD, standard deviation; LDL, low-density lipoprotein; HDL, high-density lipoprotein.

a Mean LDL and total cholesterol values are provided, although these were not included in the definition of dyslipidemia because their recommended levels vary according to other risk factors (http://www.cfpc.ca/english/cfpc/programs/patient%20education/cholesterol/default.asp).

b Independent *t* test for differences between sex, unless otherwise noted.

c Data presented as median (interquartile range) because of skewed distribution; statistical analysis with Mann-Whitney test.

d Mann-Whitney test (unequal variances).

**Table 3:** Plasma Apolipoprotein Levels by Sex and Risk Factors for Cardiovascular Disease Among 483 Canadian First Nations Adults, 2003^a^

Characteristic^b^	n (%)	ApoB (g/L)	*P* Value	ApoA1 (g/L)	*P* Value	ApoB:ApoA1 Ratio	*P* Value
**Sex**							
Men	230 (48)	0.94 (0.28)	.046	1.14 (1.03-1.23)^c^	.004^d^	0.84 (0.63-1.02)^c^	.001^d^
Women	253 (52)	0.89 (0.26)		1.17 (1.05-1.31)^c^		0.75 (0.59-0.91)^c^	
**Obese**							
Yes	265 (56)	0.97 (0.96)	<.001	1.13 (0.17)	<.001	0.87 (0.24)	<.001
No	204 (44)	0.83 (0.79)		1.20 (0.19)		0.71 (0.24)	
**At-risk waist circumference**							
Yes	313 (68)	0.96 (0.26)	<.001	1.15 (0.18)	.006	0.85 (0.25)	<.001
No	151 (32)	0.82 (0.26)		1.20 (0.18)		0.70 (0.23)	
**Diabetes**							
Yes	140 (29)	1.05 (0.29)	<.001	1.16 (0.19)	.92	0.91 (0.26)	<.001
No	343 (71)	0.86 (0.25)		1.17 (0.18)		0.75 (0.23)	
**Hypertension**							
Yes	201 (43)	0.99 (0.28)	<.001	1.18 (0.19)	.10	0.85 (0.27)	<.001
No	271 (57)	0.86 (0.25)		1.15 (0.18)		0.76 (0.23)	
**Microalbuminuria**							
Yes	94 (20)	1.01 (0.81-1.26)^c^	<.001^d^	1.15 (0.17)	.49	0.90 (0.26)	<.001
No	372 (80)	0.86 (0.68-1.05)^c^		1.17 (0.18)		0.77 (0.24)	
**Cardiometabolic risk**							
Yes	212 (45)	1.05 (0.25)	<.001	1.16 (0.19)	.54	0.93 (0.24)	<.001
No	255 (55)	0.80 (0.23)		1.17 (0.18)		0.70 (0.21)	
**Dyslipidemia**							
Yes	155 (32)	1.03 (0.90-1.19)^c^	<.001^d^	1.07 (0.98-1.18)^c^	<.001^d^	0.98 (0.23)	<.001
No	328 (68)	0.81 (0.65-1.01)^c^		1.19 (1.09-1.31)^c^		0.72 (0.22)	
**Metabolic syndrome**							
Yes	252 (53)	1.02 (0.26)	<.001	1.13 (0.17)	<.001	0.91 (0.75-1.05)^c^	<.001^d^
No	223 (47)	0.80 (0.23)		1.20 (0.18)		0.64 (0.53-0.80)^c^	

Abbreviation: apo, apolipoprotein.

a Values for apoA1, apoB, and apoB:apoA1 ratio are given as mean (standard deviation), and differences were assessed by using independent-samples *t* tests, unless otherwise noted.

b Definitions for characteristics are provided in Table 1. Data were not available for all participants for every characteristic.

c Results reported are median (interquartile range) because of skewed distribution.

d Mann-Whitney U (nonparametric) test.

**Table 4:** Plasma Apolipoprotein Levels by Sex and Age Among 481^a^ Canadian First Nations Adults, 2003

**Sex and Age, y**	Mean (SD) ApoB	*P* Value^b^	Mean (SD) ApoA1	*P* Value^b^	Mean (SD) ApoB:ApoA1 Ratio	*P* Value^b^
**Men (n = 229)**						
18-29 (n = 72)	0.79 (0.26)	<.001	1.12 (0.14)	.12	0.71 (0.25)	<.001
30-39 (n = 65)	0.95 (0.25)		1.14 (0.15)		0.85 (0.25)	
40-49 (n = 49)	1.09 (0.26)		1.13 (0.16)		0.97 (0.24)	
≥50 (n = 43)	1.01 (0.25)		1.17 (0.17)		0.88 (0.24)	
**Women (n = 252)**						
18-29 (n = 70)	0.78 (0.24)	<.001	1.15 (0.20)	.03	0.69 (0.22)	.001
30-39 (n = 78)	0.89 (0.22)		1.20 (0.17)		0.75 (0.21)	
40-49 (n = 59)	0.96 (0.27)		1.21 (0.21)		0.81 (0.25)	
≥50 (n = 45)	0.99 (0.29)		1.22 (0.22)		0.83 (0.28)	

Abbreviations: SD, standard deviation; apo, apolipoprotein.

a For 2 participants, the blood sample was insufficient to assess apoA1 and apoB; priority was given to measuring the other plasma lipids (total cholesterol, low-density lipoprotein cholesterol, high-density lipoprotein cholesterol, and triglyceride).

b Analysis of variance with linear contrast.

**Table 5:** Canadian First Nations Adults at Risk for Cardiovascular Disease by Plasma Apolipoprotein Levels, 2003 (N = 481)^a^

**Sex and Age, y**	n (%) ApoB	*P* Value^b^	n (%) ApoA1	*P* Value^b^	n (%) ApoB:ApoA1 Ratio	*P* Value^b^
**Men (n = 229)**						
18-29 (n = 72)	5 (7)	.003	25 (35)	.87	20 (29)	<.001
30-39 (n = 65)	10 (15)		24 (37)		37 (57)	
40-49 (n = 49)	15 (31)		18 (37)		40 (82)	
≥50 (n = 43)	10 (23)		14 (33)		25 (58)	
**Women (n = 252)**						
18-29 (n = 70)	5 (7)	.006	52 (74)	.006	30 (43)	.01
30-39 (n = 78)	6 (8)		43 (55)		45 (58)	
40-49 (n = 59)	10 (17)		37 (63)		38 (65)	
≥50 (n = 45)	10 (22)		20 (44)		29 (64)	

Abbreviation: apo, apolipoprotein.

a Cutoffs for increased cardiovascular risk are shown in Table 1. For 2 participants, the blood sample was insufficient to assess apoA1 and apoB; priority was given to measuring the other plasma lipids (total cholesterol, low-density lipoprotein cholesterol, high-density lipoprotein cholesterol, and triglyceride).

b χ^2^ Test with linear association.
